# Development of a Recombinant O/CATHAY Topotype Foot-and-Mouth Disease Virus as a Resource for Vaccine Matching and Efficacy Evaluation

**DOI:** 10.3390/v18091019

**Published:** 2026-09-15

**Authors:** Yerin Kim, Yeonrae Chae, Yoon-Hee Lee, Seo-Yong Lee, Dong-Wan Kim, Tae-jun Kim, Ye-Ji Yun, Sung-Han Park, Sang Hyun Park, Jong-Hyeon Park, Jeong-In Baek, Hyejin Kim

**Affiliations:** 1 Center for Foot-and-Mouth Disease Vaccine Research, Animal and Plant Quarantine Agency, 177, Hyeo-ksin 8-ro, Gimcheon-si 39660, Gyeongsangbuk-do, Republic of Korea; yerin3531@korea.kr (Y.K.); codusfo1@korea.kr (Y.C.); lyhee74@korea.kr (Y.-H.L.); happyhusband0406@korea.kr (S.-Y.L.); dongwan12@korea.kr (D.-W.K.); taejun0526@korea.kr (T.-j.K.); shpark1124@korea.kr (S.-H.P.); shpark0205@korea.kr (S.H.P.); parkjhvet@korea.kr (J.-H.P.); 2 Department of Companion Animal Health, College of Rehabilitation and Health, Daegu Haany University, Gyeongsan-si 38610, Gyeongsangbuk-do, Republic of Korea; 3 Avian Disease Division, Animal and Plant Quarantine Agency, 177, Hyeoksin 8-ro, Gimcheon-si 39660, Gyeongsangbuk-do, Republic of Korea; yunyeji@korea.kr

**Keywords:** foot-and-mouth disease virus, FMDV, O/CATHAY, recombinant virus

## Abstract

Foot-and-mouth disease virus serotype O remains a global threat, particularly the O/CATHAY topotype in East and Southeast Asia. Because timely access to emerging field isolates can be limited, reverse genetics provides a flexible approach for generating viruses representing selected circulating strains for vaccine matching and efficacy evaluation. Here, we generated O/CATH/APQA-V18 and evaluated its suitability for vaccine assessment in pigs. A contemporary Vietnamese O/CATHAY strain was selected based on viral protein-1 phylogenetic analysis, and its P1 capsid-coding region was incorporated into an O1 Manisa backbone using reverse genetics. The recombinant virus was characterized by real-time reverse transcription polymerase chain reaction, transmission electron microscopy, and sequence analysis. Two-dimensional virus neutralization tests using sera against O/PanAsia-2 and O/Primorsky yielded antigenic relationship values of 0.04 and 0.11, respectively, indicating poor antigenic matching. In vivo, O/CATH/APQA-V18 induced clinical signs in unvaccinated pigs, confirming its infectivity and pathogenicity. Vaccinated pigs had low neutralizing antibody titers against O/CATH/APQA-V18 before challenge, and only 25% were protected. These findings support O/CATH/APQA-V18 as a genetically defined and reproducible experimental virus for vaccine matching and protective efficacy evaluation against contemporary O/CATHAY viruses.

## 1. Introduction

Foot-and-mouth disease (FMD) poses a serious threat to livestock production because of its rapid transmission among cloven-hoofed species, including cattle, pigs, sheep, and goats. Outbreaks can result in considerable economic losses owing to the extensive control measures and disease management efforts required to contain the spread of the disease [1,2]. The World Organisation for Animal Health (WOAH) classifies FMD as a transboundary animal disease of major economic importance [3,4,5].

FMD virus (FMDV), the causative agent of FMD, possesses a positive-sense, single-stranded ribonucleic acid (RNA) genome of approximately 8.4 kb and is classified within the genus *Aphthovirus* of the family *Picornaviridae* [5,6]. The FMDV genome encodes four structural proteins (VP1, VP2, VP3, and VP4), which constitute the icosahedral capsid, as well as multiple non-structural proteins [7,8,9]. FMDV comprises seven antigenically distinct serotypes (O, A, C, Asia1, SAT1, SAT2, and SAT3), which are further divided into topotypes according to their geographical distribution and genetic lineages based on nucleotide sequence variation. Cross-protection between serotypes is minimal, and continuous genetic and antigenic evolution even within the same serotype results in the emergence of diverse topotypes, thereby limiting cross-protective immunity [10,11,12,13].

Serotype O is the predominant FMDV serotype worldwide [14]. Among its genetically distinct topotypes, the O/CATHAY topotype has been increasingly reported in East and Southeast Asia, particularly in Vietnam, Hong Kong, and China. O/CATHAY viruses exhibit enhanced pathogenicity and transmissibility in pigs and possess distinct antigenic characteristics, primarily related to variations in the VP1 protein [15,16]. Therefore, there is an urgent need for continuous surveillance of circulating O/CATHAY viruses and the establishment of systems for evaluating vaccine efficacy targeting this lineage. Antigenic matching between vaccine strains and circulating field viruses is a critical determinant of vaccine efficacy. According to studies conducted by the World Reference Laboratory for Foot-and-Mouth Disease (WRLFMD), currently available serotype O vaccine strains, including O 3039, O/Primorsky, O/PanAsia-2, and O/Boeun, exhibit poor antigenic matching with O/CATHAY viruses, with r_1_ values < 0.3. These findings suggest that existing vaccines provide limited protection against some viruses belonging to this topotype [17]. Consequently, there is a need not only to develop novel vaccine candidates against the O/CATHAY topotype but also to establish reliable challenge models for evaluating vaccine-induced protection.

The protective efficacy of FMD vaccines must ultimately be assessed by conducting challenge studies in target animal species, which require the use of well-characterized challenge viruses with confirmed infectivity and pathogenicity [18,19,20]. In addition, the repeated use of imported field isolates is associated with considerable constraints related to biosafety, virus acquisition, and long-term maintenance. Therefore, the construction of recombinant challenge viruses based on the currently circulating epidemic strains would provide a stable and reproducible platform for evaluating vaccine efficacy in target animals.

Therefore, in this study, we generated a recombinant virus representing the O/CATHAY topotype that has been circulating in Southeast Asia in recent years and characterized its biological properties to evaluate its potential as a candidate virus for vaccine efficacy studies in pigs. Furthermore, viral shedding and virus-neutralizing antibody responses were analyzed to evaluate the suitability of the recombinant virus as a challenge model. This study provides a practical platform for evaluating vaccine efficacy against FMDV O/CATHAY and baseline data that would be valuable for future preclinical and target-animal efficacy studies of novel vaccine candidates. The recombinant virus that we generated remained genetically stable following in vivo passage and can serve as a genetically defined, reproducible challenge virus for vaccine evaluation against contemporary O/CATHAY strains.

## 2. Materials and Methods

### 2.1. Cells

Baby hamster kidney cells expressing T7 RNA polymerase (BHK T7-9; RIKEN BRC CELL BANK RCB4942, Tsukuba, Japan) and fetal porcine kidney cells (LF-BK; provided by the Agricultural Research Service, U.S. Department of Agriculture, Greenport, NY, USA) were maintained in Dulbecco’s Modified Eagle Medium (DMEM; Corning, Manassas, VA, USA). The culture medium contained 10% heat-inactivated fetal bovine serum (Gibco, Grand Island, NY, USA) and 1% penicillin–streptomycin (Corning). Cells were maintained at 37 °C with 5% CO_2_ under humidified conditions [5].

### 2.2. Phylogenetic Analysis

Phylogenetic analysis was performed using the VP1 nucleotide sequences of representative serotype O/CATHAY topotype FMDV strains retrieved from the GenBank database. Nucleotide sequences were aligned in MEGA version 11 (Molecular Evolutionary Genetics Analysis), which was subsequently used for phylogenetic reconstruction under the maximum-likelihood framework with the Tamura–Nei model. Node support was assessed using 1000 bootstrap replicates, and evolutionary distances along the branches are expressed as substitutions per nucleotide site [21].

### 2.3. Recombinant FMDV

The recombinant virus was constructed by substituting selected nucleotide sequences in the O1 Manisa vaccine strain [22]. The P1 sequence of O/VIT/18-5490/2018 (GenBank accession no. MN250316.1), a porcine O/CATHAY virus collected in Bình Phước Province, Vietnam, in 2018, was selected as the donor sequence [23,24]. VP1-based phylogenetic analysis confirmed that this strain clustered with contemporary Vietnamese O/CATHAY viruses [25]. Cloning procedures were performed by Serentec (Daejeon, Republic of Korea). For recombinant virus production, the infectious clone plasmid was transfected into BHK T7-9 cells. For transfection, plasmid deoxyribonucleic acid (DNA) and FuGENE^®^ HD Transfection Reagent (Promega, Madison, WI, USA) were mixed in Opti-MEM (Corning) at a DNA-to-reagent ratio of 1:3, and the solution was added to BHK T7-9 cells. Following transfection, the cells were transferred to a biosafety level 3 (BSL-3) facility and maintained at 37 °C for 48 h [26]. The recovered recombinant virus was designated O/CATH/APQA-V18.

To confirm the serotype of the recombinant virus, an antigen-detection assay was performed using the VDRG^®^ FMDV 3Diff/PAN Ag Rapid Kit (Median Diagnostics, Chuncheon, Republic of Korea). The kit consists of a pan-serotype test line (PAN); three serotype-specific test lines for FMDV serotypes O, A, and Asia1 (3Diff); a control line (C); and a serotype O-specific test line (O).

### 2.4. Nucleotide Sequence Analysis

The genetic identity of the recombinant virus was confirmed through nucleotide sequence analysis. Viral RNA extraction was carried out with the RNeasy Mini Kit (Qiagen, Hilden, Germany) according to the supplied protocol. Complementary DNA was generated with the SuperScript™ IV First-Strand Synthesis System (Invitrogen, Thermo Fisher Scientific, Waltham, MA, USA). Three primer pairs were designed for the amplification of the target regions (Table 1). Polymerase chain reaction (PCR) amplification was carried out using AccuPower^®^ ProFi Taq PCR PreMix (Bioneer, Daejeon, Republic of Korea). The PCR products were purified using ExoSAP-IT™ PCR Product Cleanup Reagent (Applied Biosystems, Thermo Fisher Scientific) and subjected to Sanger sequencing by Macrogen Inc. (Seoul, Republic of Korea). The obtained nucleotide sequences were assembled and analyzed using BioEdit software (version 7.2.5) [27].

### 2.5. Production and Purification of Recombinant FMDV Antigen

Following transfection of BHK-T7-9 cells, the recovered recombinant virus was serially passaged five times in LF-BK cells. BEI was prepared by cyclization of 2-bromoethylamine hydrobromide under alkaline conditions. For chemical inactivation, BEI was added to the virus suspension at a final concentration of 3 mM, followed by incubation at 26 °C for 16 h. BEI was then added again at a final concentration of 3 mM, and the suspension was incubated under the same conditions for an additional 8 h. After inactivation, 1 M sodium thiosulfate solution (DAEJUNG, Siheung, Republic of Korea) was added at 2% (*v*/*v*), and the mixture was incubated at 26 °C for 30 min with shaking at 100 rpm to neutralize residual BEI. Complete inactivation was confirmed by testing the treated virus preparation for residual infectivity in LF-BK and BHK-21 cells. No residual infectivity was detected in either cell line. The inactivated viral antigen was concentrated using 7.5% polyethylene glycol (average molecular weight 6000; Sigma-Aldrich, St. Louis, MO, USA) and 0.5 M NaCl (Sigma-Aldrich, St. Louis, MO, USA). The concentrated antigen was subsequently purified twice via 15–45% (*w*/*v*) sucrose density gradient ultracentrifugation (4 °C, 100,000× *g*, 4 h). Following ultracentrifugation, twelve fractions were collected from the sucrose gradient. For the transmission electron microscopy (TEM) analysis, purified virus samples were applied to carbon-coated grids and negatively stained with phosphotungstic acid (Sigma-Aldrich). Viral particles were then visualized using a Hitachi H-7650 transmission electron microscope (Hitachi High-Tech, Tokyo, Japan) at Kyungpook National University.

### 2.6. Vaccine Matching

The antigenic relationship between the recombinant O/CATH/APQA-V18 virus and the O/Primorsky and O/PanAsia-2 vaccine strains was evaluated using a two-dimensional virus neutralization test (2D-VNT). Bovine vaccinated sera collected at 21 days post-vaccination (dpv) from cattle vaccinated with O/Primorsky or O/PanAsia-2 were used according to the Pirbright Institute [28]. Each bovine vaccinated serum sample consisted of pooled sera from five animals. The sera were subjected to two-fold serial dilutions and mixed with each virus before the addition of susceptible cells. The mixtures were incubated at 37 °C for 72 h. Virus-neutralizing antibody titers were assessed based on the inhibition of the cytopathic effect (CPE) and were expressed as the log_10_ reciprocal dilution of serum capable of neutralizing 100 TCID_50_ (50% tissue culture infectious dose) of the virus. The antigenic relationship between the vaccine strains and recombinant virus was determined by calculating the r_1_ value, which was defined as the ratio of the heterologous neutralizing antibody titer against the recombinant virus to the homologous neutralizing antibody titer against the corresponding vaccine strain. An r_1_ value of ≥0.3 was considered indicative of antigenic matching, whereas an r_1_ value of <0.3 was interpreted as evidence for antigenic mismatch [28].

### 2.7. Animal Experiments

The Animal BSL-3 facility of the Animal and Plant Quarantine Agency (APQA) was used for all animal experiments. Approval for the animal experiments was obtained from the Institutional Animal Care and Use Committee (IACUC) of the APQA (Approval No. 2025-1921).

### 2.8. Production of Challenge Virus

To produce the challenge virus, two 9-week-old FMDV-seronegative pigs were inoculated with the recombinant virus and monitored for clinical signs for 7 days. Skin tissue containing vesicular lesions (P1) was collected from clinically affected pigs and suspended in serum-free medium, followed by vortexing for approximately 5 min. Following centrifugation of the tissue suspension, the supernatant was collected and clarified using a 0.22-μm syringe filter. The clarified material was then inoculated into LF-BK cell cultures, followed by two passages to prepare the challenge virus stock. The final stock had an infectivity titer of 2.08 × 10^7^ TCID_50_/mL.

### 2.9. Challenge Study in Pigs

To evaluate O/CATH/APQA-V18 as a challenge virus in pigs, three FMDV-seronegative pigs and four pigs vaccinated with a commercial FMD vaccine were included in the challenge study. The vaccinated pigs received a single 2 mL dose of a commercial trivalent oil-adjuvanted vaccine by intramuscular injection. The vaccine, manufactured by Boehringer Ingelheim (BI, Bracknell, UK), contained O 3039, an O/CATHAY topotype strain, together with O_1_ Manisa (serotype O) and A_22_ Iraq (serotype A), and had a reported potency of >6 PD_50_ per dose. At 28 days post-vaccination (dpv), all pigs were challenged with O/CATH/APQA-V18 by intradermal inoculation into the heel bulb. Each pig received 0.1 mL of a virus preparation containing 1 × 10^6^ TCID_50_/mL, corresponding to an administered dose of 1 × 10^5^ TCID_50_ per animal [29]. Following this challenge, clinical signs were monitored daily for 7 days, and oral and nasal swab samples were collected daily. Clinical scores were determined based on the presence of vesicular lesions on the tongue, gingiva/lips, snout, and each of the four feet (right forelimb, left forelimb, right hindlimb, and left hindlimb). One point was assigned for each affected site, and the maximum clinical score was seven. Lesions confined to the heel-bulb inoculation site were recorded as primary lesions and excluded from the clinical score. A clinical lesion was defined as an intact vesicle or a ruptured vesicle with subsequent crust formation. Pigs with no lesions at sites other than the inoculation site during the 7-day observation period were classified as protected. Pigs showing one or more lesions on the tongue, gingiva/lips, snout, or at sites on the feet other than the inoculation site were classified as unprotected. Serum samples were obtained at 0, 1, 3, 5, and 7 days post-challenge (dpc) to evaluate virus-neutralizing antibody responses. Serum was separated via centrifugation, heat-inactivated at 56 °C for 30 min, and stored at −70 °C.

### 2.10. Enzyme-Linked Immunosorbent Assay (ELISA)

Antibodies against FMDV serotype O in heat-inactivated serum samples were detected using the VDPro^®^ FMDV Type O Ab b-ELISA kit (MEDIAN Diagnostics) as instructed by the manufacturer. The optical density (OD) was measured at 450 nm, and the sample-to-negative control (*S/N*) ratio was calculated as follows:

$$S/N=\frac{OD\;of\;sample-mean\;OD\;of\;the\;positive\;control}{mean\;OD\;of\;the\;negative\;control-mean\;OD\;of\;the\;positive\;control}$$

According to the manufacturer’s instructions, samples with *S/N* values ≤ 0.60 were considered positive, whereas samples with *S/N* values > 0.60 were considered negative. For graphical presentation, ELISA results were expressed as 1 − *S/N* values such that higher values represented stronger antibody responses.

### 2.11. Virus Titration and Virus Neutralization Testing (VNT)

The WOAH Manual of Diagnostic Tests and Vaccines for Terrestrial Animals served as the reference for virus titration and VNT [30]. Virus titers were determined via endpoint titration using LF-BK cells and calculated using the Spearman–Kärber method. Viral infectivity was expressed as TCID_50_/mL. Ten-fold serial dilutions of the virus samples (10^−1^–10^−8^) were prepared in serum-free medium and inoculated into 96-well plates at 50 μL per well in duplicate. Following incubation at 37 °C in a 5% CO_2_ incubator for 2–3 days, CPEs were examined microscopically, and the 50% endpoint was determined.

The test viruses used in the VNT were O 3039 and O/CATH/APQA-V18. The viruses were propagated in LF-BK cells, and virus stocks with titers of approximately 10^7^ TCID_50_/mL were used. Heat-inactivated serum samples were initially diluted at a ratio of 1:8 in DMEM supplemented with 1% penicillin–streptomycin, followed by two-fold serial dilutions. Equal volumes of each serum dilution and test virus stock containing 100 TCID_50_ were mixed and incubated at 37 °C for 1 h. The serum–virus mixtures were added to LF-BK cell monolayers and maintained at 37 °C under 5% CO_2_ for 2–3 days. The CPEs were subsequently assessed by microscopic examination. Neutralizing antibody titers were calculated using the Spearman–Kärber method and expressed as log_10_ values [30,31].

### 2.12. Real-Time Reverse Transcription PCR (RT-PCR)

Oral and nasal swab samples collected from each animal at the same sampling point were pooled before RNA extraction. Viral RNA was extracted from the pooled samples using the HiQ Viral DNA/RNA Kit v3.0 (BioD, Gwangmyeong, Republic of Korea) according to the manufacturer’s instructions. Real-time reverse transcription PCR (RT-qPCR) was performed using the AccuPower^®^ FMDV Real-Time RT-PCR MasterMix Kit (Bioneer, Daejeon, Republic of Korea) on a CFX96 Touch™ Real-Time PCR Detection System (Bio-Rad Laboratories, Hercules, CA, USA), as previously described [32]. The primers and probe supplied with the kit were used according to the manufacturer’s instructions. For quantification, a standard curve was generated using 10-fold serial dilutions of O/CATH/APQA-V18 ranging from 1 × 10^1^ to 1 × 10^5^ viral RNA copies per 0.1 mL. The standard curve was described by the equation Cq = −3.8645log_10_(X) + 35.646, with an R^2^ value of 0.997, where X represents the viral RNA copy number per 0.1 mL. Viral RNA copy numbers in the pooled swab samples were calculated using the equation X = 10^(35.646-Cq)/3.8645)^.

### 2.13. Statistical Analysis

Data are expressed as the mean ± standard error of the mean (SEM). Statistical analyses were conducted in GraphPad Prism 8.0 (GraphPad Software, Boston, MA, USA). Group differences were examined by two-way analysis of variance (ANOVA), followed by Tukey’s or Sidak’s multiple-comparison test, as appropriate. Statistical significance was defined as *p* < 0.05. Significance levels are indicated by * *p* < 0.05, ** *p* < 0.01, *** *p* < 0.001, and **** *p* < 0.0001; ns indicates no significant difference [20].

## 3. Results

### 3.1. Phylogenetic Analysis of the O/CATHAY Topotype of FMDV

**Figure 1:**
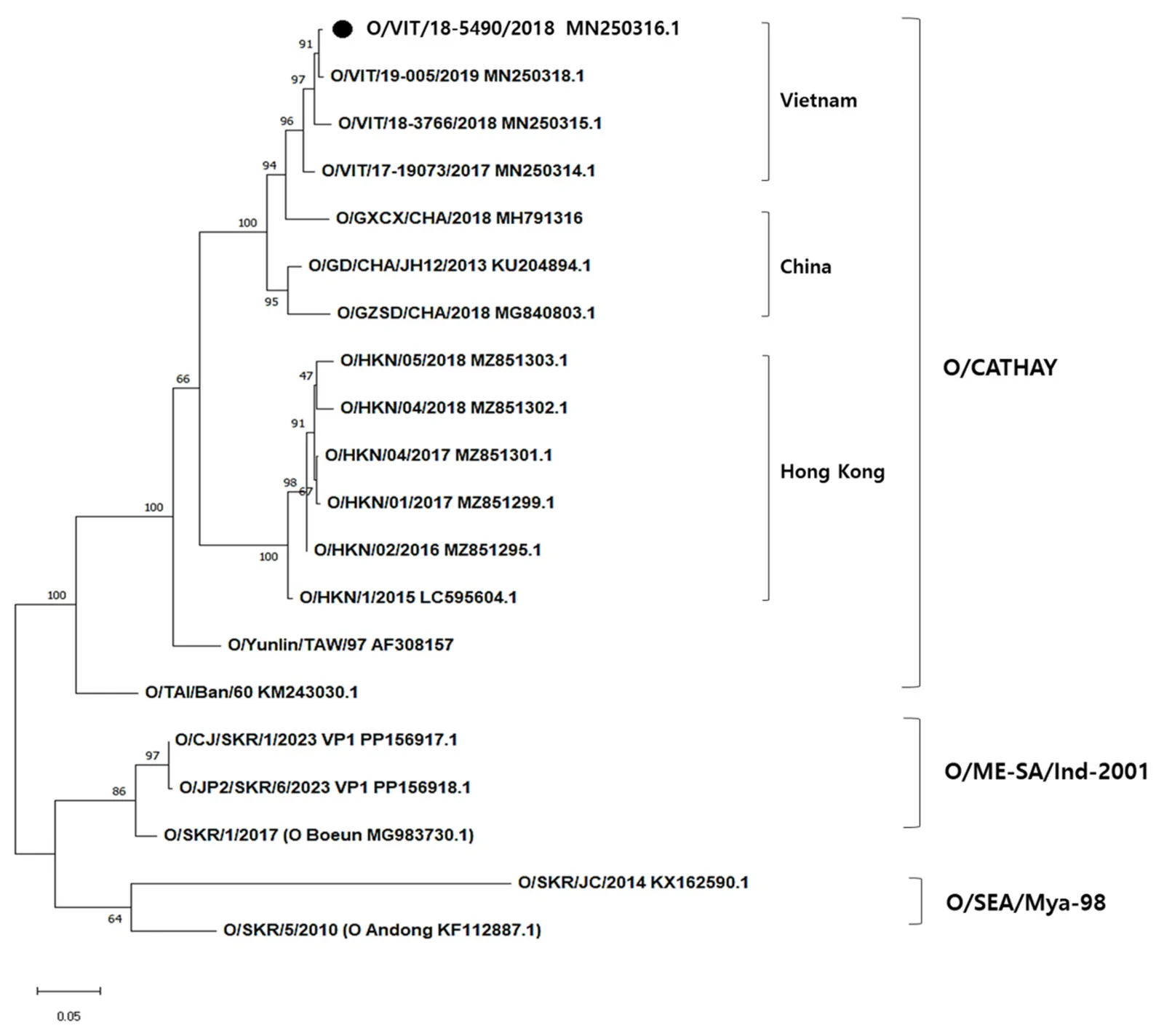
Phylogenetic analysis based on the VP1 nucleotide sequences of foot-and-mouth disease virus serotype O. Phylogenetic relationships were inferred by the maximum-likelihood method under the Tamura–Nei model. Node support was assessed using 1000 bootstrap replicates, with the resulting values shown at the corresponding branches. Branch lengths reflect nucleotide substitutions per site across the 633 positions included in the final dataset (scale, 0.05 substitutions/site). The analysis included O/CATHAY topotype viruses isolated from Hong Kong, Vietnam, and China, as well as the O/ME-SA/Ind-2001 and O/SEA/Mya-98 topotype viruses that have emerged in the Republic of Korea. This analysis was conducted using MEGA11. The O/VIT/18-5490/2018 strain, which was used as the donor of the P1 region for constructing the recombinant virus, is indicated by a filled circle (●).

To elucidate the genetic relationships of a representative FMDV of the O/CATHAY topotype circulating in East and Southeast Asia, a phylogenetic tree was constructed based on the VP1 nucleotide sequences (Figure 1). The O/CATHAY viruses were distinctly segregated from the O/ME-SA (Ind-2001) and O/SEA (Mya-98) topotypes. Within the O/CATHAY topotype, recent isolates from Vietnam and China formed a distinct cluster, whereas the Hong Kong isolates formed a separate sub-lineage. Furthermore, the vaccine strains, O/TAI/Ban60 (O 3039) and O/Yunlin/TAQ/97 (O/Taiwan97), were positioned on a basal branch separate from the recent Vietnam-China cluster, while O/VIT/18-5490/2018 clustered within the Vietnamese isolate group. Therefore, in this study, we generated a recombinant virus based on the O/VIT/18-5490/2018 strain, which was selected through comparative analysis of complete P1 sequences and represents the genetic characteristics of the contemporary O/CATHAY topotype.

### 3.2. Construction and Production of the Recombinant O/CATHAY Virus

**Figure 2:**
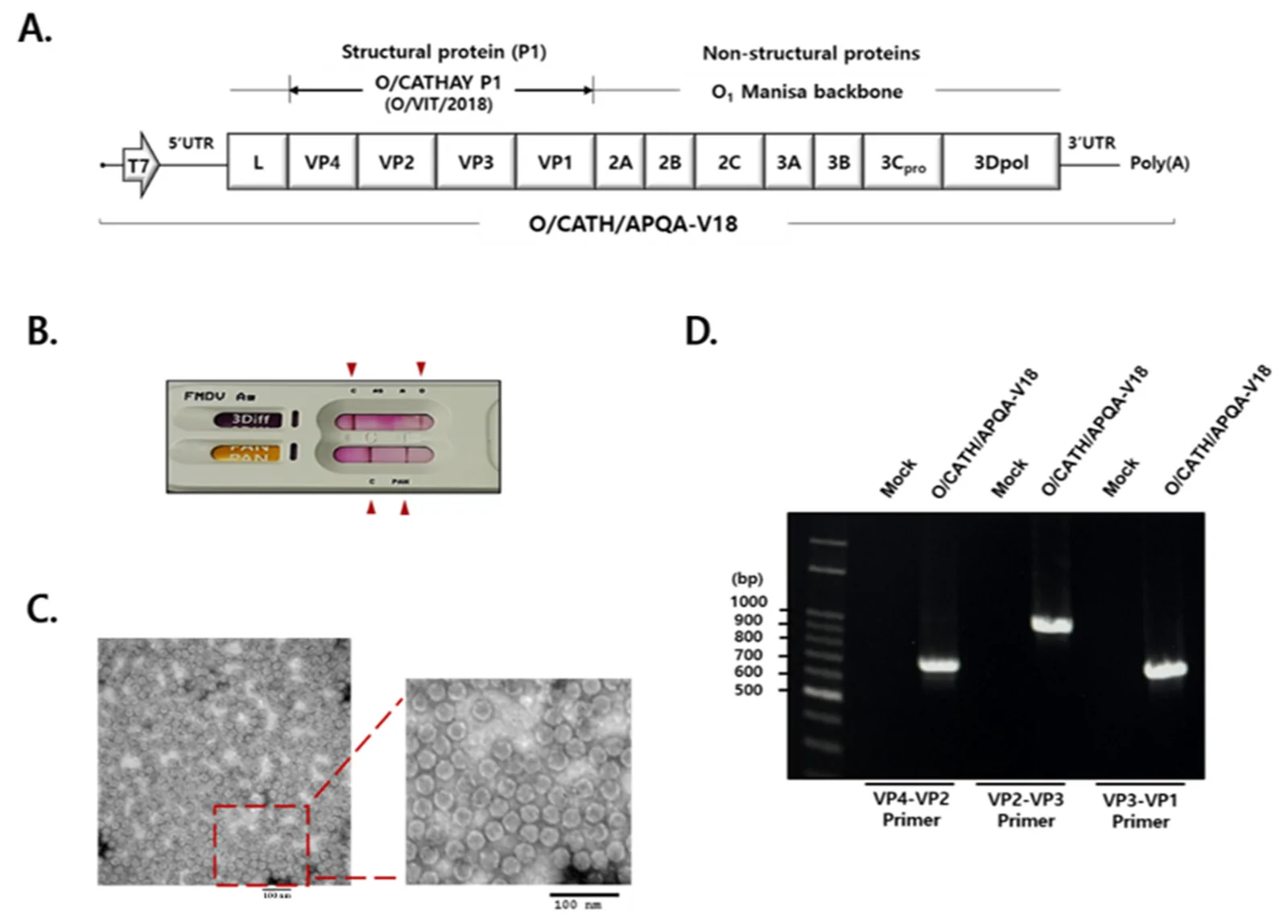
Characterization of the recombinant O/CATHAY virus, O/CATH/APQA-V18. (**A**) Schematic diagram of the recombinant viral genome comprising the O1 Manisa backbone, including the region upstream of P1, and the P1 (VP4–VP2–VP3–VP1) region derived from O/VIT/18-5490/2018. (**B**) Antigen-detection strip test results of the produced virus. (**C**) Transmission electron micrograph (TEM) of purified recombinant O/CATH/APQA-V18 virus particles. The left panel shows an overview of the purified viral particles, and the right panel represents an enlarged view of the red-boxed area. Scale bar = 100 nm. (**D**) Reverse transcription-polymerase chain reaction (RT-PCR) amplification of the structural protein-coding regions VP4–VP2, VP2–VP3, and VP3–VP1. Mock, RNA extracted from the culture supernatant of uninfected cells was used as a negative control.

To generate a stable challenge virus for the O/CATHAY topotype, a chimeric recombinant plasmid was constructed by inserting the P1 (VP4-VP2-VP3-VP1) capsid region of the O/VIT/18-5490/2018 (O/VIT/2018) strain into the O1 Manisa backbone containing a T7 promoter (Figure 2A). Subsequently, the recombinant virus was successfully produced using the T7 transcription system, and its recovery was confirmed using an antigen-detection strip assay, which showed clear positive reactions at both the control (C) and serotype O-specific test (O) lines (Figure 2B). Furthermore, TEM observations confirmed the presence of typical FMDV particles with a size of approximately 25–30 nm, confirming the successful production and structural integrity of the recombinant virus (Figure 2C). Target regions (VP4-VP2, VP2-VP3, and VP3-VP1) were successfully amplified using RT-PCR (Figure 2D), and sequence chromatograms confirmed the precise genetic identity and sequence integrity of the recombinant construct relative to the reference strains.

### 3.3. Antigenic Mismatch Analysis with Commercial Vaccine Strains

A 2D-VNT was performed to evaluate the antigenic relatedness between the O/CATHAY topotype virus and the tested commercial FMD vaccine strains. To determine the r_1_ values, bovine sera were used in the assays according to the WOAH manual. The r_1_ values against the O/PanAsia-2 (ME-SA topotype) and O/Primorsky (SEA topotype) vaccine strains were 0.04 and 0.11, respectively. Because an r_1_ value < 0.3 indicates an antigenic mismatch, these results demonstrated poor antigenic matching between the tested commercial vaccines and the O/CATHAY topotype virus, highlighting the potential need for a representative challenge virus (Table 2).

### 3.4. Sequence Characterization and Evaluation of Vaccine Efficacy Against the Recombinant O/CATH/APQA-V18 Strain

To assess sequence variation following in vivo replication and subsequent cell passage, the virus (P1) was re-isolated from the tissues of a pig that exhibited clinical signs after challenge and subsequently passaged in cells (L). The entire capsid-coding region was sequenced after the first and second cell passages (P1L1 and P1L2, respectively). Both P1L1 and P1L2 retained the capsid-coding sequence of the original recombinant virus, with the exception of a single amino acid substitution (L80M) in VP2.

**Figure 3:**
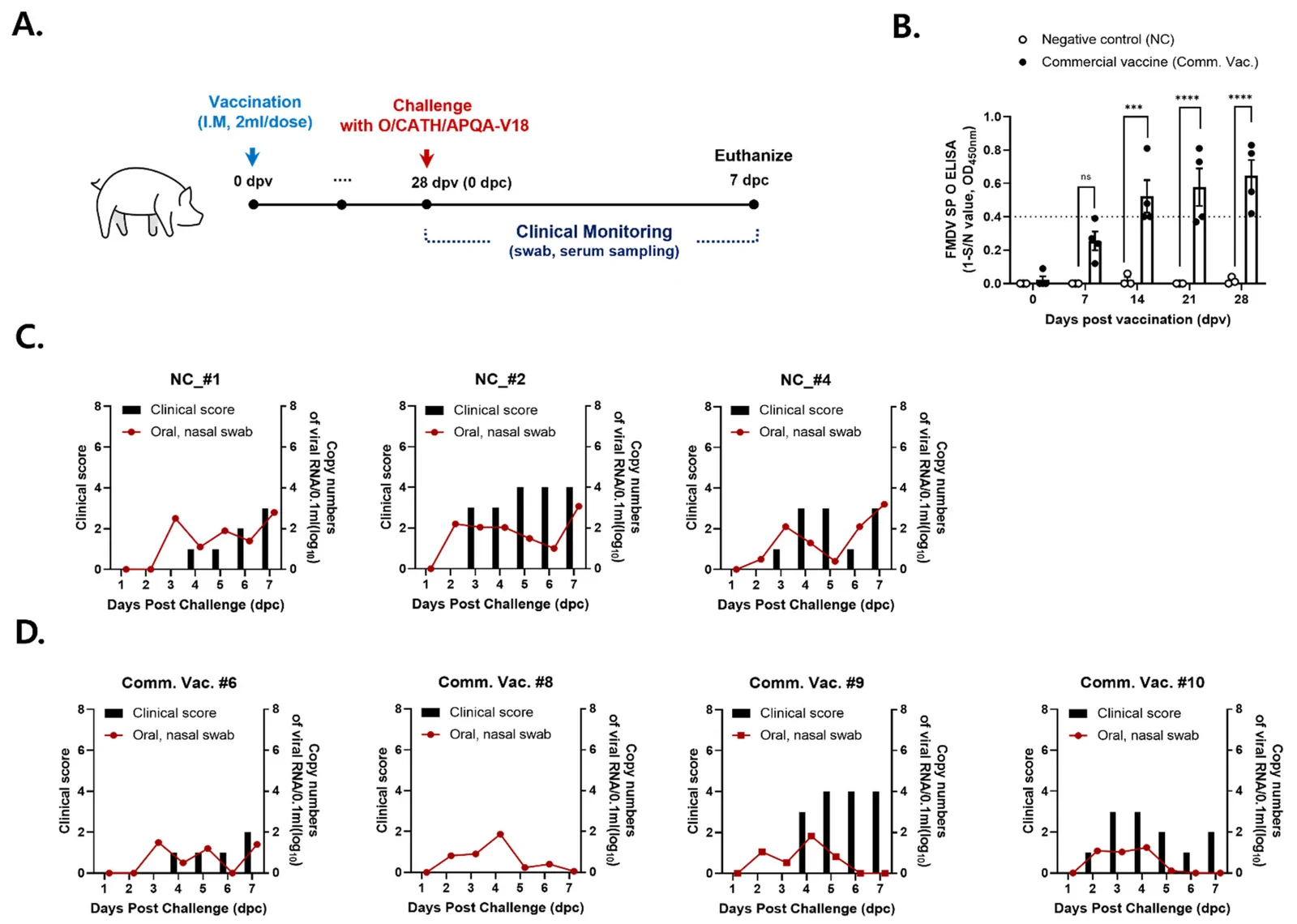
Evaluation of vaccine efficacy against the challenge virus in pigs. (**A**) Schematic timeline of the animal experiment, detailing vaccination at 0 dpv, challenge with porcine O/CATH/APQA-V18 at 28 dpv (0 dpc), daily clinical monitoring, serum and oral swab sampling, and euthanasia at 7 dpc. The ellipsis indicates the omitted time interval between 0 and 28 dpv. (**B**) FMDV type O SP enzyme-linked immunosorbent assay antibody levels (1 − *S/N* values) in the negative control (NC) and commercial vaccine (Comm. Vac.) groups. *** *p* < 0.001, and **** *p* < 0.0001 versus the control group; ns, not significant. (**C**,**D**) Individual clinical scores and viral copy numbers in oral, nasal swabs from individual pigs in the NC and Comm. Vac. groups following challenge over the 7-dpc observation period.

This experiment was conducted to determine the suitability of the porcine O/CATH/APQA-V18 strain as a challenge virus. Additionally, we evaluated whether the commercial vaccine containing the O 3039 strain (O/CATHAY lineage) provided cross-protection against recent O/CATHAY lineage viruses, in accordance with the experimental schedule (Figure 3A). Serum antibody responses, quantified via FMDV type O SP ELISA (1 − *S/N* values), were significantly elevated in the commercial vaccine group during the 28-day period following immunization (0 to 28 dpv) before challenge, whereas baseline levels were consistently maintained in the negative control group (Figure 3B). Following the viral challenge, animals in the negative control group (NC #1, #2, and #4) developed typical FMD clinical symptoms, accompanied by detectable viral shedding in oral swabs throughout the 7-dpc observation period (Figure 3C). By contrast, the commercial vaccine-immunized group (Comm. Vac. #6, #8, #9, and #10) showed a protection rate of only 25% (1/4), with three pigs remaining unprotected and exhibiting clinical symptoms and viral shedding, while one pig was successfully protected (Figure 3D).

### 3.5. Neutralization Antibody Responses to the Viral Challenge

**Figure 4:**
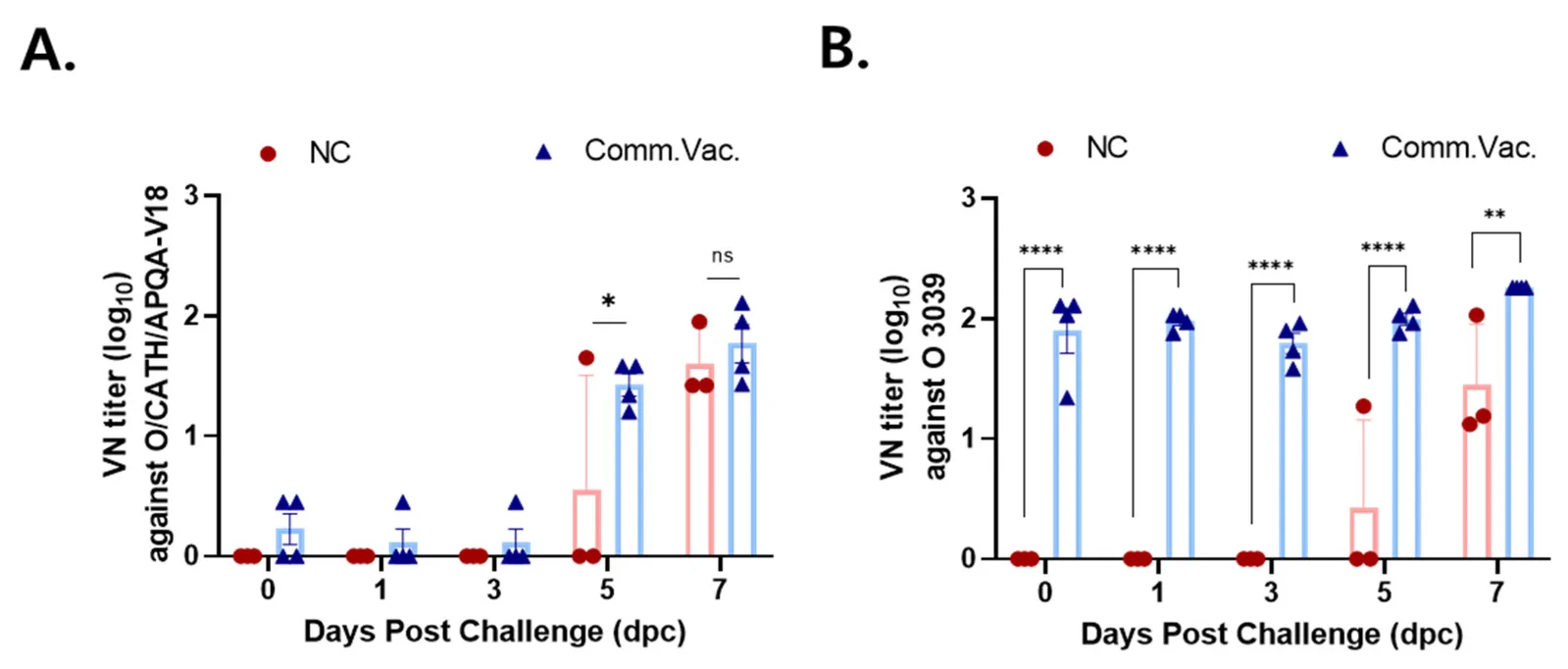
Serum neutralizing antibody responses and cross-reactivity following the viral challenge. Serum virus neutralization (VN) titers in the negative control (NC; red circles) and commercial vaccine (Comm. Vac.; blue triangles) groups over the 7-dpc monitoring period. (**A**) VN titers against the recombinant challenge strain, O/CATH/APQA-V18. (**B**) VN titers against the commercial vaccine strain, O 3039. Data are presented as means with individual data points. * *p* < 0.05, ** *p* < 0.01, and **** *p* < 0.0001 versus the control group; ns, not significant.

To evaluate humoral immune responses and cross-reactivity against O/CATHAY lineage viruses, serum virus neutralization (VN) titers against both the challenge strain, O/CATH/APQA-V18, and the commercial vaccine strain, O 3039, were measured after the viral challenge (Figure 4). In the NC group, VN titers against both strains were generally undetectable or remained at baseline levels until 3 dpc, with a subsequent modest increase observed at 5 and 7 dpc due to active viral replication (Figure 4A,B). By contrast, the Comm. Vac. group maintained consistently high pre-existing VN titers against the vaccine strain, O 3039, from 0 to 7 dpc, which were significantly higher than those in the NC group (Figure 4B). Regarding the recombinant challenge strain, O/CATH/APQA-V18, VN titers in the Comm. Vac. group were minimal on 0 dpc, indicating a lack of pre-existing neutralizing antibodies against this variant; however, these titers significantly increased by 5 dpc compared to those in the NC group, eventually reaching comparable levels by 7 dpc due to secondary immune stimulation following infection (Figure 4A).

## 4. Discussion

In this study, the recombinant FMDV O/CATHAY strain, O/CATH/APQA-V18, was successfully generated using an O1 Manisa-based reverse genetics system. The establishment of this genetically defined recombinant virus provides a reproducible challenge virus for antigenic characterization and evaluation of vaccine efficacy against contemporary O/CATHAY viruses. Unlike independently isolated field viruses, which may exhibit genetic variations between isolates or passages, the reverse genetics-derived virus provides a standardized experimental platform for reproducible vaccine evaluation [11,33,34]. Furthermore, because timely access to field isolates may be limited, this approach enables the rapid generation of viruses representing selected circulating strains when sequence information is available, thereby facilitating vaccine matching and efficacy evaluation [35,36,37]. The protective efficacy of a commercial vaccine containing O 3039 was evaluated in vaccinated pigs and unvaccinated controls. O 3039 was used as the homologous virus to measure vaccine-induced neutralizing antibody responses, which were then compared with those against O/CATH/APQA-V18. Vaccinated pigs developed high neutralizing antibody titers against O 3039 but substantially lower titers against O/CATH/APQA-V18, and only one of four animals was protected following challenge. A direct 2D-VNT comparison could not be performed because bovine reference serum against O 3039 was not available. However, reports from the WRLFMD and WOAH reference laboratories have described poor antigenic matching between contemporary O/CATHAY viruses and O 3039 [16,38,39,40]. This reported antigenic difference, together with the neutralizing antibody responses observed in the present study, may partly explain the low protection rate. A direct 2D-VNT using homologous reference serum will be required to confirm the antigenic relationship between O 3039 and O/CATH/APQA-V18.

Following in vivo passage, only a single amino acid substitution (VP2 L80M) was identified in the capsid-coding region. Leucine and methionine are both hydrophobic amino acids with similar physicochemical properties, making L80M a relatively conservative substitution that would not generally be expected to cause major destabilization of the overall capsid structure [41]. However, the functional consequence of an amino acid substitution depends on the structural and functional context of the affected residue. VP2 residue 80 has previously been associated with cell culture adaptation in combination with VP1 E83K [42,43], suggesting that substitutions at this position may influence the viral phenotype in the presence of additional capsid mutations. However, VP1 E83K was not detected in the present study. Therefore, although major structural destabilization due to VP2 L80M alone may be unlikely, a site-specific functional effect cannot be excluded. The biological significance of VP2 L80M therefore remains unclear, and further studies are needed to determine whether this substitution affects viral replication, pathogenicity, or antigenic properties. Because sequence analysis was limited to the capsid-coding region of virus recovered from a single animal, whole-genome sequencing of viruses from multiple animals and passages will be required to assess the genetic consistency of the recombinant virus.

Interestingly, sera collected from non-vaccinated pigs following the O/CATH/APQA-V18 challenge also exhibited neutralizing activity against O 3039. This finding suggests that infection with O/CATH/APQA-V18 induced antibodies capable of cross-neutralizing O 3039. However, the mechanism underlying this cross-reactivity remains unknown and warrants further investigation. The limited protection demonstrated in this study agrees with recent reports showing that currently used commercial FMD vaccines fail to provide adequate protection against contemporary O/CATHAY viruses, even after a two-dose vaccination regimen administered at a two-week interval [19]. Collectively, these findings highlight the urgent need to develop vaccine strains that are more closely matched to currently circulating O/CATHAY viruses.

In this context, genetically defined challenge viruses are essential tools for vaccine matching and the reliable evaluation of vaccine efficacy during vaccine development [3,33]. The recombinant O/CATH/APQA-V18 established in this study is a stable and reproducible challenge virus that can facilitate antigenic characterization, vaccine matching, and preclinical evaluation of next-generation vaccine candidates. Thus, the recombinant virus developed in this study offers a practical experimental system to support the assessment and optimization of vaccines targeting currently circulating O/CATHAY viruses.

## 5. Conclusions

In conclusion, this study established the recombinant FMDV O/CATHAY strain, O/CATH/APQA-V18. The virus remained genetically stable following in vivo passage and can serve as a genetically defined, reproducible challenge virus for the evaluation of vaccines against contemporary O/CATHAY strains. Our findings underscore the need for improved vaccines with better antigenic matching to currently circulating O/CATHAY viruses. The recombinant virus provides a practical platform for antigenic characterization, vaccine matching, and preclinical evaluation of next-generation vaccine candidates, thereby facilitating the development and evaluation of improved vaccines against emerging O/CATHAY viruses.

## Figures and Tables

**Table 1:** Primers used for sequence analysis of the recombinant virus.

Name	Primer Pair	Sequence (5’ → 3’)
VP4-VP2	L_F	AACCGCTTAACGGAGAGTGG
	VP2_R	AGTGATGTGTGCCGTCATGT
VP2-VP3	VP2_F	TGCCAACTGACCACAAAGGT
	VP3_R	GTCAACCGGCAAGCGCAAGT
VP3-VP1	VP3_F	TCCAGATAACGCACGGGAAG
	2A_R	ATCTCCCGCCAATTTGAGCA

F, Forward primer; R, Reverse primer.

**Table 2:** Antigenic relationships (r_1_ values) between the recombinant O/CATHAY virus and the tested commercial vaccine strains determined using a 2D-VNT.

Vaccine Strain	Topotype	r_1_ Value *	Matching Status
O/PanAsia-2	O/ME-SA	0.04	N ^†^
O/Primorsky	O/SEA	0.11	N

* An r_1_ value < 0.3 indicates antigenic mismatch. ^†^ Antigenic mismatch with the vaccine strain (N).

## Data Availability

The data presented in this study are available in the article.

## Abbreviations

The following abbreviations are used in this manuscript:

FMD	foot-and-mouth disease
WOAH	World Organisation for Animal Health
FMDV	foot-and-mouth disease virus
RNA	ribonucleic acid
WRLFMD	World Reference Laboratory for Foot-and-Mouth Disease
BHK T7-9	baby hamster kidney cells expressing T7 RNA polymerase
LF-BK	fetal porcine kidney cells
DMEM	Dulbecco’s Modified Eagle Medium
DNA	deoxyribonucleic acid
BSL-3	biosafety level 3
PCR	polymerase chain reaction
BEI	binary ethyleneimine
TEM	transmission electron microscopy
2D-VNT	two-dimensional virus neutralization test
dpv	days post-vaccination
CPE	cytopathic effect
TCID_50_	50% tissue culture infectious dose
dpc	days post-challenge
ELISA	enzyme-linked immunosorbent assay
*S/N*	sample-to-negative control
RT-PCR	real-time reverse transcription polymerase chain reaction
VN	virus neutralization
VNT	virus neutralization testing
VP 1-4	viral proteins 1-4
MEGA	molecular evolutionary genetics analysis
NC	negative control
Comm. Vac.	commercial vaccine
r_1_	antigenic relationship value
IACUC	Institutional Animal Care and Use Committee
APQA	Animal and Plant Quarantine Agency
SEM	standard error of mean
ANOVA	analysis of variance
PAN	pan-serotype test line
